# An International Hospital Congress

**Published:** 1912-09-28

**Authors:** 


### The Hospital

The Workers' Newspaper of Administrative Medicine and Institutional Life, Administration, National Insurance and Health.

No. 1269, Vol. LII. SATURDAY, SEPTEMBER 28, 1912. PRICE ONE PENNY.

### AN INTERNATIONAL HOSPITAL CONGRESS.

The organ of the Canadian Hospital Association, the *Hospital World*, pleads strongly for the holding of an International Hospital Congress. With that plea we are in cordial agreement. During recent years the principal hospital countries of the civilised world have been at pains to organise their experts. They have successfully founded Hospital Associations, which are really authoritative societies who may claim to speak with no uncertain voice in the discussion of matters that affect local hospital politics. Here in England we have our own British Hospitals Association, of which this journal is the official organ. In the United States flourishes the American Hospital Association, with a membership of nearly a thousand, and the *International Hospital Record* as its official journal. In Canada reigns the flourishing Canadian Hospital Association, whose official organ is the *Hospital World*; in Holland is the new association whose views are voiced in *Het Ziekenhuis*; in Germany, the Verein der leitenden Verwaltungsbeambten von Krankenanstalten, whose journal is the well-known *Zeitschrift*; while in Italy, in France, and in Belgium there exist smaller societies with the same objects and similar ideals. In a word, hospital effort and opinion are organised, though they are not yet everywhere in touch. What is wanted to complete the solidarity and promote international co-operation is a common platform. Congresses, notwithstanding their comparatively high percentage of dead-head members, play an important part in developing the subjects with which they deal.

They bring together men and women who have common interests, but who often possess different methods of exploiting these interests. They enable a process of comparison and contrast to be instituted, and therefore conduce to a more instructive understanding of ways and means that are unfamiliar to one section though perfectly well known to another. As educative factors it is difficult to overestimate their value or importance: as factors in promoting social good feeling, international amity, and professional co-operation between the various countries their usefulness has been fully realised in recent times. Next year we hold in London the Thirteenth International Congress of Medicine—an event that will focus the attention of the lay as well as of the professional public upon the Metropolis. But medicine can appeal to only a small section. But with hospitals the case is widely different. To the layman they are of vital interest, whether they are voluntary institutions supported by the free gifts of the charitable, or hospitals managed, financed, and controlled by the ratepayers, the provision for which comes from the rates. Nor must it be forgotten that in modern days hospital politics include subjects which are all embracing. They range from Poor-Law administration and the methods of dealing with particular phases of illness, poverty, or destitution to large general principles of sociology.

We in this country hold staunchly by our voluntary system. Abroad they know little about it, less of the way in which it works, of its advantages and difficulties, and of its splendid past and glorious present. What we have to tell our foreign colleagues on these points must be instructive and useful. As our contemporary, the *Hospital World*, rightly remarks, there are many other points which can be discussed at the Congress. "Let the German Director tell how care-free he is from financial embarrassment; why he builds such elaborate baths and disinfection houses; why he builds the one-storey pavilion among trees and gardens; and how he provides for teaching medical students and for medical research. Let the Britisher tell the story of the munificent gifts to establish such wonderful hospitals as old St. Bart.'s, Guy's, King's College, St. Thomas's; of the work of Lister, Addison, Graves, and Bright in these old haunts; of their homely comfortable wards, and their great special funds, and of their troubles with Insurance Bills . . . and let the American tell of the phenomenal growth of hospitals in this new land, of their splendid schools for nursing, of their ingenious apparatus, and of the fine work rendered by their distinguished members. . . . Such a Congress would call the attention of the world to hospital work, hospital progress, and hospital needs. It would stimulate philanthropy, encourage hospital workers, and be of an untold benefit, indirectly, to the millions of sick folk who are obliged to seek the care of hospitals."

We heartily endorse all this, and echo our contemporary's conclusion, "Let us have a Congress." Surely England, as the mother of the modern hospital, is fit to take the lead in this matter. Our Association at its next Council meeting might well consider the subject, and elect a small provisional committee to report on it, with a view to making arrangements in the near future for holding such a Congress in London. We know the question of expense stands in the way, but it is an obstruction which is not likely to persist when the intention of holding such an international gathering has been made public, and an appeal for support, backed by responsible hospital workers, has been issued. Let us at least have a committee to investigate the pros and cons. The time is favourable for discussing the matter, even if it be held wiser, in view of next year's Congress of Medicine, to postpone the Hospital meeting until a later date.

